# Optimization of an Efficient Semi-Solid Culture Protocol for Sterilization and Plant Regeneration of *Centella asiatica* (L.) as a Medicinal Herb

**DOI:** 10.3390/molecules16118981

**Published:** 2011-10-25

**Authors:** Sina Siavash Moghaddam, Hawa Binti Jaafar, Maheran Abdul Aziz, Rusli Ibrahim, Asmah Bt Rahmat, Elizabeth Philip

**Affiliations:** 1 University Putra Malaysia, 43400 UPM Serdang, Selangor, Malaysia; Email: drhawazej.postgrads@gmail.com (H.B.J.); maheran@agri.upm.edu.my (M.A.A.); asmah@medic.upm.edu.my (A.B.R.); 2 Agrotechnology & Biosciences Division, Malaysian Nuclear Agency, Bangi 43000 Kajang, Selangor, Malaysia; Email: rusli_ibrahim@nuclearmalaysia.gov.my; 3 Forestry and Environment Division, Forest Research Institute Malaysia (FRIM), Kepong, 52109 Kuala Lumpur, Malaysia; Email: philip@frim.gov.my

**Keywords:** *C.asiatica*, semi-solid culture, sterilization, PPM

## Abstract

The present study investigates the effects of different concentrations, as well as type of plant growth regulators (PGRs) and medium (MS, Duchefa) on the growth and development of *Centella asiatica* in semi-solid culture. In addition, a protocol for successful sterilization of *C.asiatica* explants prepared from field-grown plants highly exposed to fungal and bacterial contamination was determined. Results for sterilization treatments revealed that applying HgCl_2_ and Plant Preservative Mixture (PPM) with cetrimide, bavistin and trimethoprim which were included after washing with tap water, followed by the addition of PPM in the medium, produced a very satisfactory result (clean culture 90 ± 1.33%) and TS5 (decon + cetrimide 1% + bavistin 150 mg/L + trimethoprim 50 mg/L + HgCl_2_ 0.1% + PPM 2% soak and 2 mL/L in medium) was hence chosen as the best method of sterilization for *C.asiatica.* The synergistic combination of 6 benzylaminopurine (BAP) and 1-naphthaleneacetic acid (NAA) in concentrations of 2 mg/L and 0.1 mg/L, respectively, in Duchefa medium compared with MS induced the most optimal percentage of sprouted shoots (93 ± 0.667), number of shoots (5.2 ± 0.079) and nodes (4 ± 0.067) per explant, leaf per explant (14 ± 0.107) and shoot length (4.1 ± 0.67 cm). Furthermore, optimum rooting frequency (95.2 ± 0.81%), the number of roots/shoot (7.5 ± 0.107) and the mean root length (4.5 ± 0.133 cm) occurred for shoots that were cultured on full-strength MS medium containing 0.5 mg/L indole-3-butyric acid (IBA). In this study, the acclimatized plantlets were successfully established with almost 85% survival. The findings of this study have proven an efficient medium and PGR concentration for the mass propagation of *C.asiatica*. These findings would be useful in micropropagation and *ex situ* conservation of this plant.

## 1. Introduction

*Centella asiatica* is a valuable medicinal and aromatic herb which is spread throughout the tropical and sub-tropical regions. It has its origin in the wetlands of Asia, such as in China, India, and Malaysia. It contains a number of triterpene saponins (e.g., asiaticoside), sapogenins, glycosides, alkaloids (hydrocotylin) and flavonoids with therapeutical properties [1]. The demand for *C.asiatica* is now met from the natural population, which has led to its gradual reduction. Tissue culture techniques can play a significant role in the rapid multiplication of elite genotypes and germplasm conservation of *C.asiatica.* Meanwhile, *in vitro* plant regeneration has been conducted in *C.asiatica* via callus culture from leaf explants [2] and somatic embryos [3]. Previous study by Karthikeyan *et al.* [4] on nodal segments of *C.asiatica* found that BAP and IBA are appropriate for shooting and rooting, respectively. In this study, a procedure for high frequency of plant regeneration from nodal segments is described.

The experimental aims of these investigations were to examine the effects of different concentrations, as well as type of plant growth regulators (PGRs) and medium on the growth and development of *C.asiatica*. Moreover, previous research involving *C.asiatica* revealed a high percentage of contamination during the culture establishment stage [5], so this study also determined a protocol for successful sterilization of *C.asiatica* explants prepared from field-grown plants that are typically highly contaminated with fungus and bacteria.

Nowadays, the most common method employed for the micropropagation of *C.asiatica* involves the propagation of shoots *via* a solid or semi-solid system. Such a semi-solid system has successfully contributed to improved multiplication yields, and it has become gradually more important in improving productivity and reducing the time taken to multiply commercially important material.

## 2. Results and Discussion

### 2.1. Sterilization Protocol

**Figure 1:**
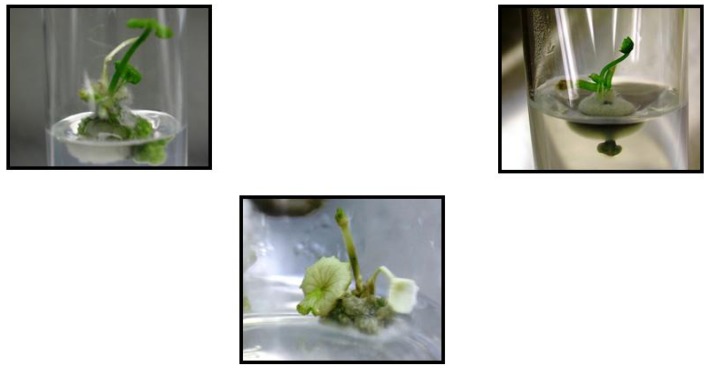
Contaminated nodal cultures without using PPM and HgCl_2_.

**Figure 2:**
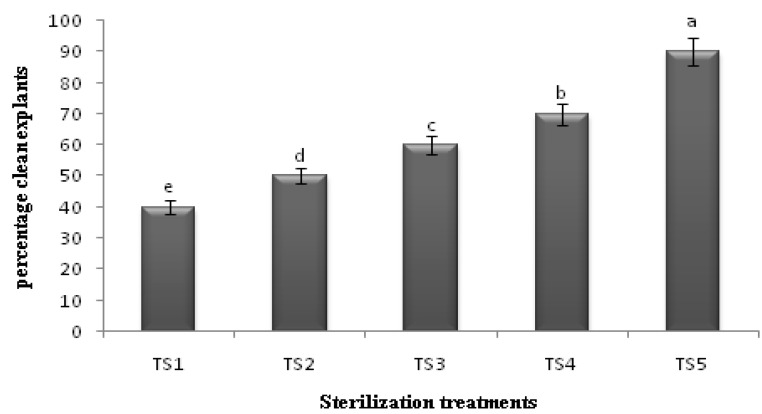
Effects of different surface sterilization methods on percentage of clean culture of *C.asiatica* n = 4 (**TS1** = decon + benomyl (dip explants 20 min) + clorox 15% and 10% (15 and 10 min respectively); **TS2** = TS1 + benomyl 100 mg/L in medium; **TS3** = Decon + benomyl + Clorox 15%, 12 min + PPM in medium 2 mL/L; **TS4** = TS3 + soak in PPM 2% for 1 hour; **TS5** = Decon + cetrimide 1% + bavistin 150 mg/L + trimethoprim 50 mg/L + HgCl_2_ 0.1% + PPM 2% soak and 2 mL/L in medium.

Prior to the initiation of culture, the nodal explants were collected from well-established field-grown plants. The sterilization procedures that were initially followed (TS1 and TS2) did not include the use of HgCl_2_ and PPM treatments, therefore a high percentage of the explants (60 ± 2.15%) was found to be contaminated (Figure 1). However, HgCl_2_ and PPM with cetrimide, bavistin and trimethoprim were included after washing with tap water, followed by the addition of PPM in the medium, to give a very satisfactory result (clean culture 90 ± 1.33%) and (TS5) was hence chosen as the best method of sterilization for *C.asiatica* (Figure 2). Tiwari *et al.* [4] reported that the initiation of the *C.asiatica* nodal culture proved to be difficult due to heavy fungal and bacterial contaminations. Thus, the nodal explants were first soaked in a solution of systemic fungicide (bavistin) and antibiotic (trimethoprim) to avoid heavy contamination, and subsequently, nearly 80% of the cultures were contamination-free. However, it was also found that the duration of the treatment for mercuric chloride is very critical due to the soft, herbaceous nature of the explants. Meanwhile, Patra *et al.* [6] stated that 20 minutes treatment with mercuric chloride would not only cause blackening of the tissues but also subsequent death of the explants. Hence, a limited treatment of 3–4 minutes of mercuric chloride was employed in this study. Joshee *et al.* [7] reported that a 60 min soak in a 2% PPM solution before culture establishment and all culture media supplemented with 2 mL/L PPM helped to control fungal and bacterial contaminations.

### 2.2. Aseptic Culture Establishment

#### 2.2.1. Semi-Solid Culture Method

**Figure 3:**
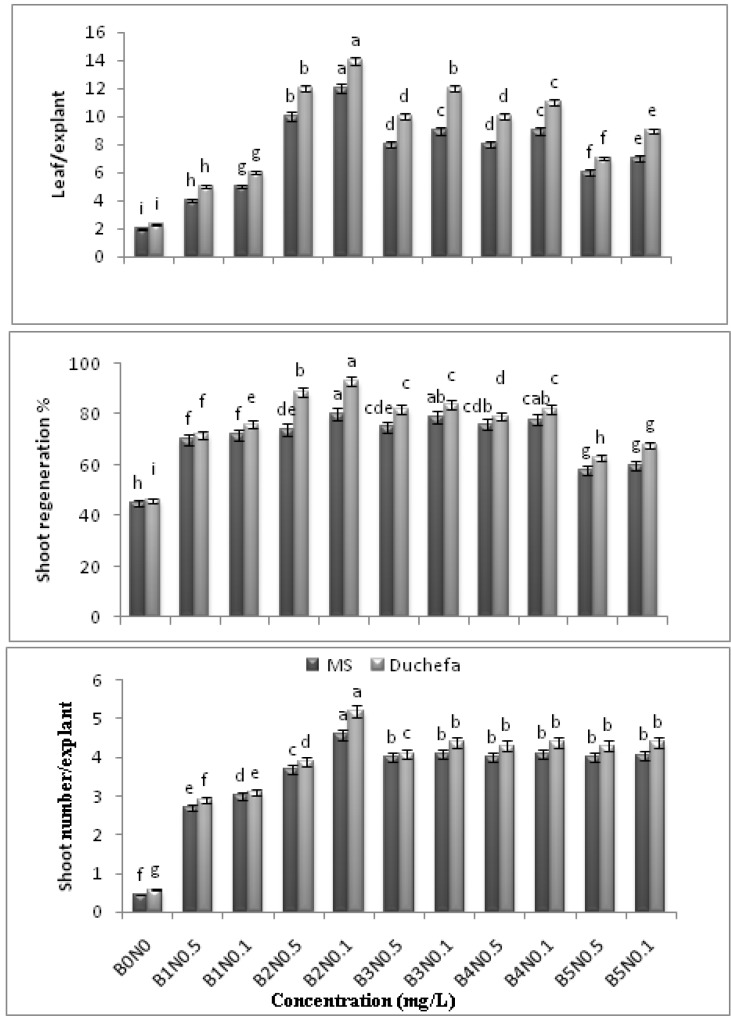
Effects of BAP and NAA concentrations in MS and Duchefa semi-solid media on number of shoots per explant, number of explants responding to form shoot and leaf per explant in *C.asiatica* (BAP = B, NAA = N, MS = Murashige and Skoog, Duchefa = Duchefa shoot media n = 4 (Example: B2N0.1 = BAP 2 mg/L and NAA 0.1 mg/L).

**Figure 4:**
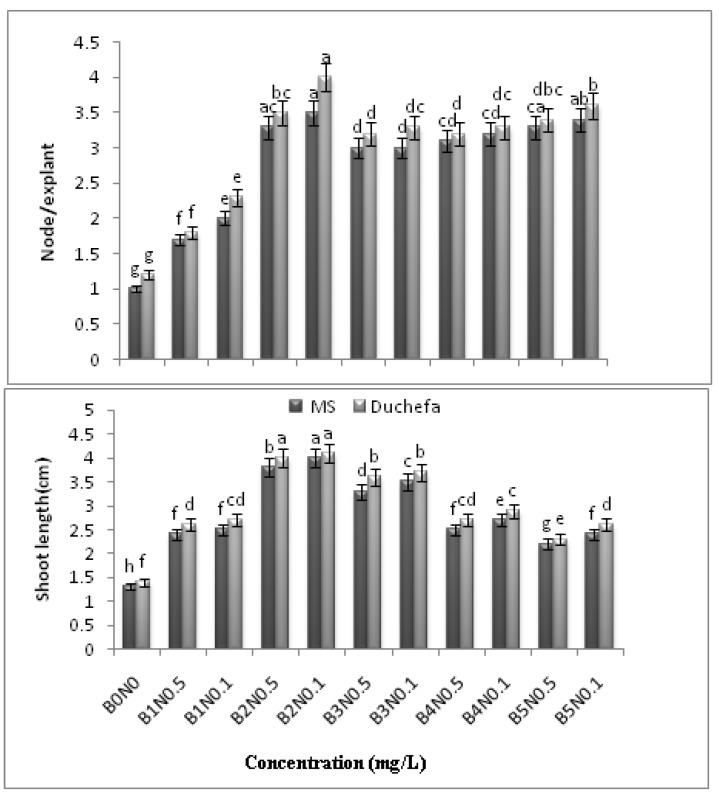
Effects of BAP and NAA concentrations in MS and Duchefa semi-solid media on number of node/explant and shoot length in *C.asiatica* n = 4 (BAP = B, NAA = N, MS = Murashige and Skoog, Duchefa = Duchefa shoot media (Example: B2N0.1 = BAP 2 mg/L and NAA 0.1 mg/L).

Various media formulation and plant growth regulators, such as auxin and cytokinin, were assessed to regenerate shoots from the nodal segments of *C.asiatica* (Figure 3 and Figure 4). Bud break did not occur during the initial 6–7 days after inoculation. However, the bud breaks started in most of the cultures from days 11–12, and the small sprouted buds then proliferated into fully expanded shoots with leaves within 3–4 weeks.

Meanwhile, various responses and significant differences were observed for the shoot proliferation from the nodes in full-strength MS and Duchefa semi-solid medium that were supplemented with different concentrations of BAP and NAA after 3 weeks of culture. Although bud breaks were observed in all the media assessed in the present study, based on the visual observation treatment (B2N0.1) in Duchefa medium was found to be the optimal combination in terms of the percentage of sprouted shoot (93 ± 0.667), number of shoots (5.2 ± 0.079) and nodes (4 ± 0.067) per explant, leaf per explant (14 ± 0.107) and shoot length (4.1 ± 0.67 cm). Meanwhile, MS supplemented with (B2N0.1) recorded for the node/explant (3.5 ± 0.067), leaf/explant (12 ± 0.067) and shoot length (4 ± 0.067 cm). Nonetheless, there was no significant difference in terms of the shoot length in the full-strength MS and Duchefa semi-solid medium containing (B2N0.1) (Figure 3 and Figure 4).

**Figure 5:**
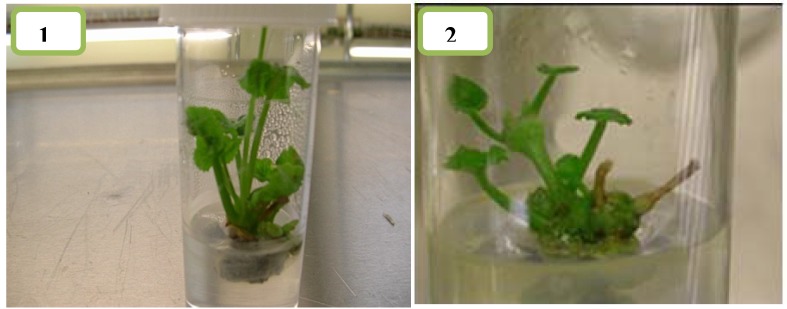
Shoot regeneration in MS (1) and Duchefa shoot medium (2) supplemented with **(B2N0.1)** in *C.asiatica* after 3 weeks.

The findings of the current study are consistent with those reported by Sharma [8] and Karthikeyan *et al.* [4] (using BAP maximum 2–3 mg/L), but they contradict the reports published by Tiwari *et al.* [5] (using 5 mg/L BAP). In fact, the nodal segments that were cultured on the MS and Duchefa media showed bud break, expansion of nodes and leaf/explant, as well as an increase in the length of the shoots. At the same time, the frequency of the responding explants was found to rapidly increase with the increase in the BAP concentration up to 2 mg/L + NAA 0.1 mg/L. Meanwhile, further increase in the level of both the phytohormones resulted in the formation of callus. Based on the results, the development of shoot regeneration (%), shoot number, leaf/explant, node/explant and shoot length are shown as the effects of different concentrations of BAP and NAA, as well as the medium formulation (Figure 5). Meanwhile, there were no significant differences found between the means of the *in vitro* response of shoots in the different MS and Duchefa semi-solid media without PGR (B0N0) (MS= 45 ± 1.07; Duchefa= 46 ± 0.66).

The two types of plant growth regulator applied in the study are cytokinins (BAP) and auxins (NAA, IBA). Cytokinins are derived from adenine and create two instant effects on undifferentiated cells: the stimulation of DNA synthesis and enhanced cell division. Auxins are indole or indole-like compounds that promote cell expansion, in particular, cell elongation. Auxins stimulate adventitious root development as well. In addition, light affects the physiological activity of IAA whereas synthetic auxins (such as NAA) are not as light sensitive. In the meantime, high cytokokinin to low auxin ratio promotes adventitious bud formation and overcome apical dominance [9].

### 2.3. Rooting

The analysis of variance re­vealed significant differences among the root treatments in terms of the frequency of cultures showing root regeneration, the number of roots/explant and root length in the semi-solid method. Nonetheless, there was no rooting on full and half-strength MS basal medium without auxin, but rooting was found to occur in 70–95% of the shoots when both media were supplemented with IBA. Apparently, the full-strength MS medium was better than the half-strength MS medium for root initiation.

**Figure 6:**
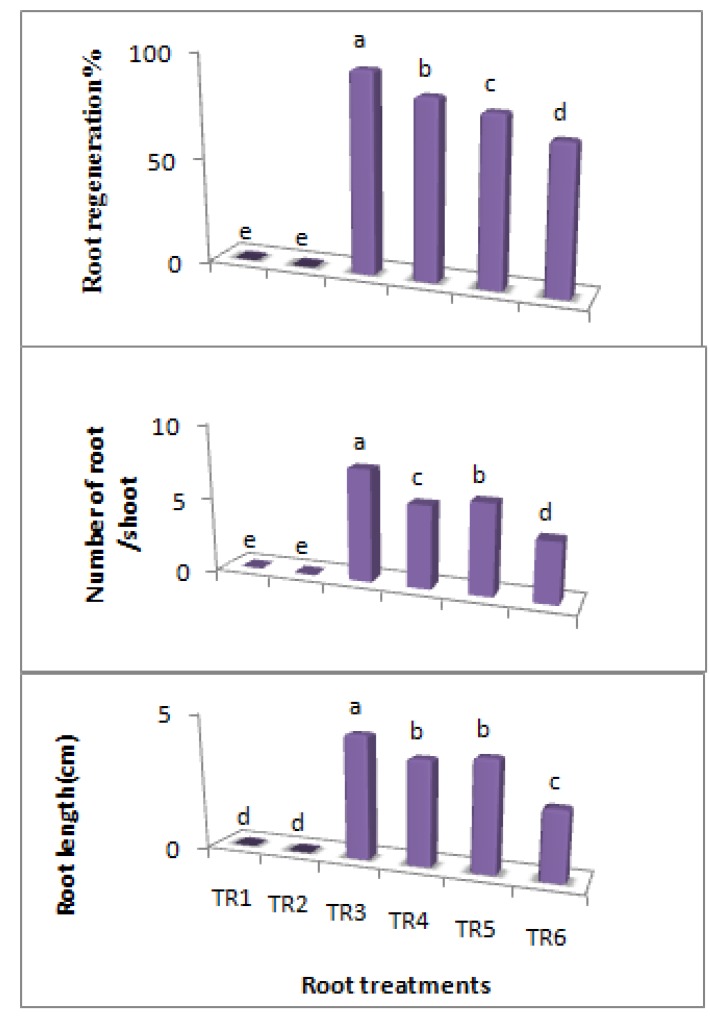
Effects of MS medium strength and IBA concentration on rooting of *in vitro* regenerated shoots of *C.asiatica.* n = 4 (TR1 = MS without IBA; TR2 = ½ MS without IBA; TR3 = MS + IBA 0.5 mg/L; TR4 = ½ MS + IBA 0.5 mg/L; TR5 = MS + IBA 0.75 mg/L; TR6 = ½ MS + IBA 0.75 mg/L).

**Figure 7:**
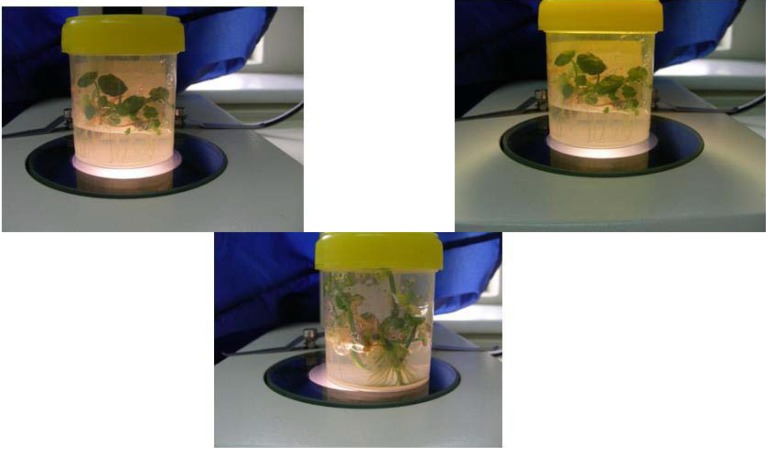
Rooting of *C.asiatica* shoots in full-strength MS medium supplemented with IBA 0.5 mg/L after 3 weeks.

A comparison using Duncan test revealed that the optimum rooting frequency (95.2 ± 0.81%), the number of roots/shoot (7.5 ± 0.107) and mean root length (4.5 ± 0.133 cm) occurred on shoots that were cultured on the full-strength MS medium containing 0.5 mg/L IBA (Figure 6 and Figure 7). Banerjee *et al.* [2] also reported the positive effect of IBA on rooting in *C.asiatica*, whereas IAA and low level of sucrose were found to be the optimum by Patra *et al.* [6].

### 2.4. Establihment of Plants in Soil

In this study, the acclimatized plantlets were successfully established with almost 85% survival. According to Tiwari *et al.* [5], the rooted plants of *C.asiatica* which were transferred from the culture tubes/flasks into plastic cups containing Soilrite had 90% survival (180 out of 200 plants), while the acclimatized plantlets were successfully established (with only 5% mortality) in the field.

Sivakumar *et al.* [10] investigated a similar series of experiments and demonstrated that for acclimatization, well-developed rooted plants were transplanted to the tray filled with soil mixture, containing Canadian sphagnum peat moss, perlite and vermiculite, which were maintained in the growth chamber for two weeks. The plantlets were then transferred into the glasshouse, potted in natural red soil for 2 more weeks. Afterwards, these potted plants were located outside, *i.e.*, under full sun, and this resulted in 95% survival.

## 3. Experimental

### 3.1. Evaluation of Different Sterilization Protocols

Preliminary experiments with *C.asiatica* showed very high contamination during the culture establishment stage. This study elaborates on the protocol for a successful sterilization of the *C.asiatica* explants, which were prepared from field-grown plants, with an abundance of fungal and bacterial contaminations. For this purpose, five treatments were applied to find the best method to be used to overcome contamination, and these are as presented in Table 1 below.

**Table 1:** Different treatments for sterilization of *C.asiatica.*

Sterilization Treatments
**TS1**= Decon + benomyl (20 min) + Clorox 15% and 10% (15 and 10 min respectively)
**TS2**= TS1 + benomyl 100 mg/L in medium
**TS3**= Decon + benomyl + Clorox 15%, 12 min + PPM in medium 2mL/L
**TS4**= TS3 + soak in PPM 2% for 1 hour
**TS5**= Decon + cetrimide 1% + bavistin 150 mg/L + trimethoprim 50 mg/L + HgCl_2_ 0.1% + PPM 2% soak and 2 mL/L in medium

TS = sterilization treatment; decon = detergent; benomyl = fungicide; clorox = bleach; HgCl_2_ = mercuric cloride; cetrimide = antiseptic agent; bavistin = fungicide; trimetoprim = antibacterial; PPM = plant preservative mixture.

To date, various methods have been developed and introduced for the sterilization of *C.asiatica* explants. In the present investigation, young shoots with nodes were collected. For sterilization treatment (TS1) the plant materials were washed with Decon, and then stirred in 1 g benomyl for 20 min. The next step was constant stirring of plant materials in 15% and 10% Clorox (sodium hypochlorite) for 15 and 10 min respectively. Treated plant materials were rinsed 3 times with autoclaved deionized water in a laminar flow cabinet. A different sterilization treatment was also employed (TS2) which included TS1 procedure as well as using benomyl (100 mg/L) in medium. Nodal explants in TS3 and TS4 were treated using PPM in medium (2 mL/L) and PPM as a soaking treatment respectively.

Sterilization treatment TS5 is descibed as follows: after removing the leaves and roots the nodal segments (as part of the stolon) were washed with detergent using an anti-bacterial sponge, and then rinsed thoroughly under running tap water. After that, nodal pieces approximately 2.5 cm in length were excised from the stolons. The nodal segments were soaked in a mixture of 1% cetrimide solution, containing 150 mg/L bavistin and 50 mg/L trimethoprim for 20 min. The explants were then surface sterilized with 0.1% (w/v) mercuric chloride for 3–4 min, followed by five rinses with autoclaved sterilized distilled water. This was followed by 60 min soaking of the explants in PPM 2% and finally the addition of 2 mL/L of PPM into the medium. Later, the nodal explants were trimmed from both ends to about 1.5 cm prior to inoculation on the culture medium.

### 3.2. Culture Medium

The culture media used in the study included the Murashige and Skoog basal medium, as well as the shoot medium from Duchefa, using semisolid culture. The composition of Duchefa shoot medium differs from the MS medium in the amount of anhydrous NaH_2_PO_4_ (128 mg/L) as one of the macroelements and thiamine HCl (0.4 mg/L) as one of the vitamines. Moreover, it only has myo-inositol and thiamine HCl as vitamins [11]. The single nodes which were separated from the nodal segment were cultured on the semisolid full-strength MS [12] and the shoot medium (Duchefa) that was supplemented with 11 different concentrations of BAP and NAA (Table 2). The shoot number per explant, node/explant, leaf/explant and shoot length was measured at the end of the 3^rd ^ week.

**Table 2:** Different PGR combinations (BAP and NAA) in semi-solid MS or Duchefa shoot media.

PGR Treatments (MS or Duchefa shoot media) mg/L
T1 = B0N0
T2 = B1N0.5
T3 = B1N0.1
T4 = B2N0.5
T5 = B2N0.1
T6 = B3N0.5
T7 = B3N0.1
T8 = B4N0.5
T9 = B4N0.1
T10 = B5N0.5
T11 = B5N0.1

BAP = B, NAA = N, MS = Murashige and Skoog, (Example: B2N0.1 = BAP 2 mg/L and NAA 0.1 mg/L).

The experiments were set up in RCBD in five replicates. There were 10 explants in each replicate. The analysis of variance (ANOVA) that is appropriate for the design was also carried out to detect the significance in terms of the differences among the means for all the treatments. The means for all the treatments were com­pared using the Duncan Multiple Range Test (DMRT) at a 5% probability level according to Gomez and Gomez [13].

Proliferating shoots were transferred to full or half-strength MS medium containing different levels of IBA for rooting (Table 3) so as to identify the optimal concentrations of the auxin and MS strength for root development after three weeks. Meanwhile, the percentage of root regeneration, number of root/shoot and root length were recorded.

**Table 3:** Rooting treatments (IBA + MS).

Treatments mg/L
TR1 = MS without IBA
TR2 = ½ MS
TR3 = MS + IBA 0.5 mg/L
TR4 = ½ MS + IBA 0.5 mg/L
TR5 =MS + IBA 0.75 mg/L
TR6 = ½ MS + IBA 0.75 mg/L

MS = Murashige and Skoog basal medium

### 3.3. Culture Conditions

The cultures were kept in an incubation room at 25 ± 2 °C, under the condition of a 16-h photoperiod of 50 µmol/m^2^/s irradiance, provided by cool white fluorescent light with 55–60% relative humidity.

## 4. Conclusions

An efficient protocol for *in vitro* propagation of the valuable medicinal plant *C.asiatica* (L.) through axil­lary shoot proliferation from nodal explants has been described. Multiple shoots were induced from the nodal segments that were cultured on semi-solid Murashige and Skoog (MS) and Duchefa shoot media containing various concentrations BAP and NAA.

A comparison of MS and Duchefa semisolid media was made and the difference in shoot multiplication was noted and apparently, the best-developed shoots were obtained using the Duchefa semi-solid medium containing B2N0.1 after three weeks. Therefore, it could be reiterated that the selection of the different types of medium and the PGR is important for optimizing the proliferation of shoots in the culture. Elongated shoots were separated and rooted on half and full-strength MS semi-solid media that were fortified with different concentrations of IBA which ranged from 0 to 0.75 mg/L. The length of roots and the number of roots/shoot were found to be the greatest in full-strength MS medium with 0.5 mg/L IBA. Moreover, about 95% of the shoots were rooted.

The micropropagation protocol standardized in the present study is established as an efficient tool for mass production of *Centella asiatica* and can be employed for its conservation. In fact, reconsidering the hypothesis postulated at the beginning of the study, it is now possible to state that the appropriate conditions for nodal segment culture of *C.asiatica* have been successfully established to cater for the continuous need of planting materials for the pharmaceutical industry. Regarding HgCl_2_, which is being widely used to overcome contamination, it should be noted that it has detrimental effects on health and causes environmental issues. Hence, in sterilization protocol HgCl_2_ could be omitted. Although more experiments are needed to find the precise concentration of PPM (as a substitute for HgCl_2_) to improve the protocol. Moreover this is the first time that Duchefa shoot media was examined on *C.asiatica* and introduced as an efficient media instead of MS. 

## References

[1] Mohd-Zainol M., Abdul-Hamid A., Abu-Bakar F., Pak-Dek S. (2009). Effect of different drying methods on the degradation of selected flavonoids in *Centella asiatica*. Int. Food Res. J..

[2] Banerjee S., Zehra M., Kumar S. (1999). *In vitro* multiplication of *Centella asiatica*, a medicinal herb from leaf explants. Curr. Sci. (Bangalore).

[3] Martin K. (2004). Plant regeneration through somatic embryogenesis in medicinally important *Centella asiatica* L. In Vitro Cell. Dev.Biol. Plant.

[4] Karthikeyan K., Chandran C., Kulothungan S. (2009). Rapid clonal multiplication through *in vitro* axillary shoot proliferation of *Centella asiatica* L. Indian J. Biotechnol..

[5] Tiwari K.N., Sharma N.C., Tiwari V., Singh B.D.  (2000). Micropropagation of *Centella asiatica* (L.), a valuable medicinal herb. Plant Cell Tiss. Organ Cult..

[6] Patra A., Rai B., Rout G., Das P.  (1998). Successful plant regeneration from callus cultures of *Centella asiatica* (linn.) urban. Plant Growth Regul..

[7] Joshee N., Biswas B.K., Yadav A.K. (2007). Somatic embryogenesis and plant development in *C.asiatica* L., a highly prized medicinal plant of the tropics*.*. HortScience.

[8] Sharma S. (2004). Micropropagation studies on *Centella asiatica* linn—An important medicinal plant. Master Dissertation.

[9] Mineo L., Goldman C.A. Plant tissue culture techniques. Tested Studies for Laboratory Teaching; Proceedings of the Eleventh Workshop/Conference of the Association for Biology Laboratory Education (ABLE), 195 pages.

[10] Sivakumar G., Alagumanian S., Rao M. (2006). High frequency *in vitro* multiplication of *Centella asiatica*: An important industrial medicinal herb. Eng. Life Sci..

[11] Huang L.C., Murashige T. (1976). Murashige & Skoog Shoot Multiplication Medium B, Plant tissue culture media major constituents, their preparartion and some applications. TCA Manual.

[12] Hung C.D., Johnson K., Torpy F. (2006). Liquid culture for efficient micropropagation of wasabia japonica (miq.) matsumura. In Vitro Cell. Dev. Biol. Plant.

[13] Gomez K.A., Gomez A.A. (1984). Statistical Procedures for Agricultural Research.

